# The Impact of Performance Expectancy, Workload, Risk, and Satisfaction on Trust in ChatGPT: Cross-Sectional Survey Analysis

**DOI:** 10.2196/55399

**Published:** 2024-05-27

**Authors:** Avishek Choudhury, Hamid Shamszare

**Affiliations:** 1 Industrial and Management Systems Engineering Benjamin M. Statler College of Engineering and Mineral Resources West Virginia University Morgantown, WV United States

**Keywords:** ChatGPT, chatbots, health care, health care decision-making, health-related decision-making, health care management, decision-making, user perception, usability, usable, usableness, usefulness, artificial intelligence, algorithms, predictive models, predictive analytics, predictive system, practical models, deep learning, cross-sectional survey

## Abstract

**Background:**

ChatGPT (OpenAI) is a powerful tool for a wide range of tasks, from entertainment and creativity to health care queries. There are potential risks and benefits associated with this technology. In the discourse concerning the deployment of ChatGPT and similar large language models, it is sensible to recommend their use primarily for tasks a human user can execute accurately. As we transition into the subsequent phase of ChatGPT deployment, establishing realistic performance expectations and understanding users’ perceptions of risk associated with its use are crucial in determining the successful integration of this artificial intelligence (AI) technology.

**Objective:**

The aim of the study is to explore how perceived workload, satisfaction, performance expectancy, and risk-benefit perception influence users’ trust in ChatGPT.

**Methods:**

A semistructured, web-based survey was conducted with 607 adults in the United States who actively use ChatGPT. The survey questions were adapted from constructs used in various models and theories such as the technology acceptance model, the theory of planned behavior, the unified theory of acceptance and use of technology, and research on trust and security in digital environments. To test our hypotheses and structural model, we used the partial least squares structural equation modeling method, a widely used approach for multivariate analysis.

**Results:**

A total of 607 people responded to our survey. A significant portion of the participants held at least a high school diploma (n=204, 33.6%), and the majority had a bachelor’s degree (n=262, 43.1%). The primary motivations for participants to use ChatGPT were for acquiring information (n=219, 36.1%), amusement (n=203, 33.4%), and addressing problems (n=135, 22.2%). Some participants used it for health-related inquiries (n=44, 7.2%), while a few others (n=6, 1%) used it for miscellaneous activities such as brainstorming, grammar verification, and blog content creation. Our model explained 64.6% of the variance in trust. Our analysis indicated a significant relationship between (1) workload and satisfaction, (2) trust and satisfaction, (3) performance expectations and trust, and (4) risk-benefit perception and trust.

**Conclusions:**

The findings underscore the importance of ensuring user-friendly design and functionality in AI-based applications to reduce workload and enhance user satisfaction, thereby increasing user trust. Future research should further explore the relationship between risk-benefit perception and trust in the context of AI chatbots.

## Introduction

ChatGPT (OpenAI) [1] is a powerful tool for a wide range of tasks, from entertainment and creativity to health care queries [2]. However, there are potential benefits associated with this technology. For instance, it can help summarize large amounts of text data [3,4] or generate programming code [5]. There is also the notion that ChatGPT may potentially assist with health care tasks [6-9]. However, the risks associated with using ChatGPT can hinder its adoption in various high-risk domains. These risks include the potential for inaccuracies and lack of citation relevance in scientific content generated by ChatGPT [10], ethical issues (copyright, attribution, plagiarism, and authorship) [11], the risk of hallucination (inaccurate information that sounds plausible scientifically) [12], and the possibility of biased content and inaccurate information due to the quality of training data sets generated prior to the year 2021 [4].

In the discourse concerning the deployment of ChatGPT and similar artificial intelligence (AI) technologies, it is sensible to recommend their use primarily for tasks a human user can execute accurately. Few studies have advocated using the technology under human supervision [13,14]. Encouraging users to rely on such tools for tasks beyond their competence is risky, as they may need help to evaluate the AI’s output effectively. The strength of ChatGPT lies in its ability to automate more straightforward, mundane tasks, freeing human users to invest their time and cognitive resources into critical tasks (not vice versa). This approach to technology use maintains a necessary balance, leveraging AI for efficiency gains while ensuring that critical decision-making remains within the purview of human expertise.

As we transition into the subsequent phase of ChatGPT deployment, establishing realistic performance expectations and understanding users’ perceptions of risk associated with its use are crucial in determining the successful integration of this AI technology. Thus, understanding users’ perceptions of ChatGPT becomes essential, as these perceptions significantly influence their usage decisions [2]. For example, suppose users believe that ChatGPT’s capabilities surpass human knowledge. In that case, they may be tempted to use it for tasks such as self-diagnosis, which could lead to potentially harmful outcomes if the generated information is mistaken or misleading. Conversely, a realistic appraisal of the limitations and strengths of technology would encourage its use in low-risk, routine tasks and foster a safer, more effective integration into our everyday lives.

Building upon the importance of user perceptions and expectations, we must also consider that the extent to which users trust ChatGPT hinges mainly on the perception of its accuracy and reliability. As users witness the technology’s ability to perform tasks effectively and generate correct, helpful information, their trust in the system grows. This, in turn, allows them to offload routine tasks to the AI and focus their energies on more complex or meaningful endeavors. Similarly, instances where the AI generates inaccurate or misleading information can quickly erode users’ perception of the technology. Users may become dissatisfied and lose trust if they perceive the technology as unreliable or potentially harmful, particularly if they have previously overestimated its capabilities. This underlines the importance of setting realistic expectations and accurately understanding the strengths and limitations of ChatGPT, which can help foster a healthy level of trust and satisfaction among users. Ultimately, establishing and maintaining trust and satisfaction are not a onetime event but an ongoing process of validating the AI’s outputs, understanding and acknowledging its limitations, and making the best use of its capabilities within a framework of informed expectations and continuous learning. This dynamic balance is pivotal for the effective and safe integration of AI technologies such as ChatGPT into various sectors of human activity.

In our prior work, we explored the impact of trust in the actual use of ChatGPT [15]. This study aims to explore a conceptual framework delving deeper into the aspects influencing user trust in ChatGPT.

**Figure 1:**
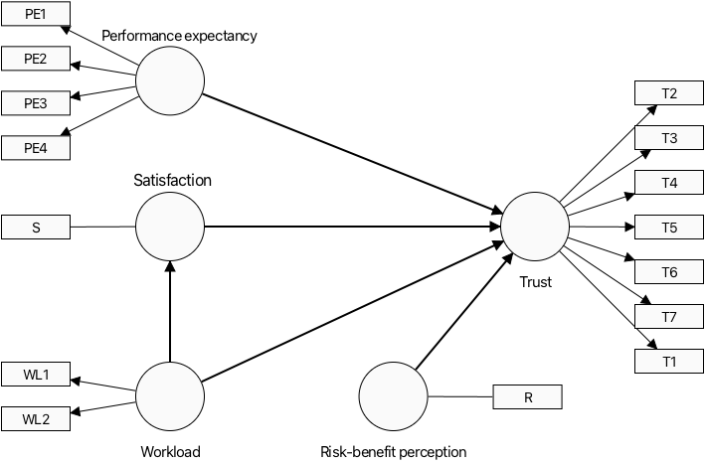
A conceptual model of technology acceptance illustrating trust (T) as the dependent outcome variable, with performance expectancy (PE), workload (WL), and risk-benefit perception (R) as direct predictors. Satisfaction (S) is depicted as a mediating variable that moderates the impact of workload on trust.

As shown in Figure 1, the proposed conceptual model is grounded in the well-established theories of technology acceptance and use, incorporating constructs such as performance expectancy, workload, satisfaction, risk-benefit perception, and trust to comprehensively evaluate user interaction with technology. Performance expectancy, derived from the core postulates of the technology acceptance model (TAM) [16] and the unified theory of acceptance and use of technology (UTAUT) [17], posits that the perceived use of the technology significantly predicts usage intentions. Workload, akin to effort expectancy, reflects the perceived cognitive and physical effort required to use the technology, where a higher workload may inversely affect user satisfaction—a construct that encapsulates the fulfillment of user expectations and needs through technology interaction. The risk-benefit perception embodies the user’s assessment of the technology’s potential advantages against its risks, intricately influencing both user satisfaction and trust. Trust, a pivotal determinant of technology acceptance [15], signifies the user’s confidence in the reliability and efficacy of the technology. This theoretical framework thus serves to elucidate the multifaceted process by which users come to accept and use a technological system, highlighting the critical role of both cognitive appraisals and affective responses in shaping the technology adoption landscape.

We explore the following hypotheses:

- Hypothesis 1: Perceived workload of using ChatGPT negatively correlates with user trust in ChatGPT.
- Hypothesis 2: Perceived workload of using ChatGPT negatively correlates with user satisfaction with ChatGPT.
- Hypothesis 3: User satisfaction with ChatGPT positively correlates with trust in ChatGPT.
- Hypothesis 4: User trust in ChatGPT is positively correlated with the performance expectancy of ChatGPT.
- Hypothesis 5: The risk-benefit perception of using ChatGPT is positively correlated with user trust in ChatGPT.

## Methods

### Ethical Considerations

The study obtained ethics approval from West Virginia University, Morgantown (protocol 2302725983). The study was performed in accordance with relevant guidelines and regulations. No identifiers were collected during the study, and all users were compensated for completing the survey through an audience paneling service. In compliance with ethical research practices, informed consent was obtained from all participants before initiating the survey. Attached to the survey was a comprehensive cover letter outlining the purpose of the study, the procedure involved, the approximate time to complete the survey, and assurances of anonymity and confidentiality. It also emphasized that participation was completely voluntary, and participants could withdraw at any time without any consequences. The cover letter also included the contact information of the researchers for any questions or concerns the participants might have regarding the study. Participants were asked to read through the cover letter information carefully and were instructed to proceed with the survey only if they understood and agreed to the terms described, effectively providing their consent to participate in the study.

### Study Design

A semistructured, web-based questionnaire was disseminated to adult individuals within the United States who engaged with ChatGPT (version 3.5) at least once per month. Data collection took place between February and March 2023. The questionnaire was crafted using Qualtrics (Qualtrics LLC), and its circulation was handled by Centiment (Centiment LLC), a provider of audience-paneling services. Centiment’s services were used due to their extensive reach and ability to connect with a diverse and representative group via their network and social media. Their fingerprinting technology, which uses IP address, device type, screen size, and cookies, was used to guarantee the uniqueness of the survey respondents. Prior to the full-scale dissemination, a soft launch was carried out with 40 responses gathered. The purpose of a soft launch, a limited-scale trial of the survey, is to pinpoint any potential problems, such as ambiguity or confusion in questions, technical mishaps, or any other factors that might affect the quality of data obtained. The survey was made available to a larger audience following the successful soft launch.

Table 1 shows the descriptive statistics of the survey questions used in this study. We developed 3 latent constructs based on the question: trust, workload, and performance expectancy, and 2 single question variables: satisfaction and risk-benefit perception. Participant responses to all the questions were captured using a 4-point Likert scale ranging from 1=strongly disagree to 4=strongly agree. These questions were adapted from constructs used in various models and theories such as the TAM, the theory of planned behavior, UTAUT, and research on trust and security in digital environments.

- Trust: Questions T1-T7 related to trust in AI systems were adapted from the trust building model [18].
- Workload: WL1 and WL2 questions from the National Aeronautics and Space Administration Task Load Index for measuring perceived workload [19].
- Performance expectancy: PE1-PE4 are about the perceived benefits of using the system, which is a central concept in TAM and UTAUT.
- Satisfaction: The single item relates to overall user satisfaction, a common measure in information systems success models [20].
- Risk-benefit perception: Question addresses the user’s assessment of benefits relative to potential risks, an aspect often discussed in the context of technology adoption and use [21].

These references provide a starting point for understanding the theoretical underpinnings of the survey used in this study. They are adapted from foundational works in information systems, human-computer interaction, and psychology that address trust, workload, performance expectancy, satisfaction, and the evaluation of benefits versus risks in technology use.

- Trust: Questions T1-T7 related to trust in AI systems were adapted from the trust building model [18].
- Workload: WL1 and WL2 questions from the National Aeronautics and Space Administration Task Load Index for measuring perceived workload [19].
- Performance expectancy: PE1-PE4 are about the perceived benefits of using the system, which is a central concept in TAM and UTAUT.
- Satisfaction: The single item relates to overall user satisfaction, a common measure in information systems success models [20].
- Risk-benefit perception: Question addresses the user’s assessment of benefits relative to potential risks, an aspect often discussed in the context of technology adoption and use [21].

These references provide a starting point for understanding the theoretical underpinnings of the survey used in this study. They are adapted from foundational works in information systems, human-computer interaction, and psychology that address trust, workload, performance expectancy, satisfaction, and the evaluation of benefits versus risks in technology use.

**Table 1:** Study variables and latent construct (N=607).

Survey items			Value, mean (SD)
**Trust (T)**			
	T1: ChatGPT is competent in providing the information and guidance I need	3.20 (0.83)	
	T2: ChatGPT is reliable in providing consistent and dependable information	3.16 (0.80)	
	T3: ChatGPT is transparent	3.12 (0.86)	
	T4: ChatGPT is trustworthy in the sense that it is dependable and credible	3.17 (0.84)	
	T5: ChatGPT will not cause harm, manipulate its responses, or create negative consequences for me	3.10 (0.88)	
	T6: ChatGPT will act with integrity and be honest with me	3.19 (0.82)	
	T7: ChatGPT is secure and protects my privacy and confidential information	3.27 (0.81)	
**Workload (WL)**			
	WL1: Using ChatGPT was mentally demanding	3.21 (0.75)	
	WL2: I had to work hard to use ChatGPT	2.20 (0.98)	
**Performance expectancy (PE)**			
	PE1: ChatGPT can help me achieve my goals	3.24 (0.77)	
	PE2: ChatGPT can reduce my workload	3.22 (0.78)	
	PE3: ChatGPT improves my work efficiency	3.21 (0.84)	
	PE4: ChatGPT helps me make informed and timely decisions	3.26 (0.79)	
**Satisfaction (S)**			
	S: I am satisfied with ChatGPT	3.24 (0.76)	
**Risk-benefit perception (R)**			
	R: The benefits of using ChatGPT outweigh any potential risks	3.20 (0.80)	

### Statistical Analysis and Model Validation

To test our hypotheses and structural model, we used the partial least squares structural equation modeling (PLS-SEM) method, a widely used approach for multivariate analysis. PLS-SEM enables the estimation of complex models with multiple constructs, indicator variables, and structural paths, without making assumptions about the data’s distribution [22]. This method is beneficial for studies with small sample sizes that involve many constructs and items [23]. PLS-SEM is a suitable method because of its flexibility and ability to allow for interaction between theory and data in exploratory research [24]. The analyses were performed using the *SEMinR* package in R (R Foundation for Statistical Computing) [25]. We started by loading the data set collected for this study using the *reader* package in R. We then defined the measurement model. This consisted of 5 composite constructs: trust, performance expectancy, workload, risk-benefit perception, and satisfaction. Trust was measured with 7 items (T1 through T7), performance expectancy with 4 items (PE1 through PE4), and workload with 2 items (WL1 and WL2), while risk-benefit perception and satisfaction were each measured with a single item. We also evaluated the convergent and discriminant validity of the latent constructs, which we assessed using 3 criteria: factor loadings (>0.50), composite reliability (>0.70), and average variance extracted (>0.50). We used the Heterotrait-Monotrait ratio (<0.90) to assess discriminant validity [26].

Next, we defined the structural model, which captured the hypothesized relationships between the constructs. The model included paths from risk-benefit perception, performance expectancy, workload, satisfaction to trust, and a path from workload to satisfaction. We then estimated the model’s parameters using the partial least squares method. This was done with the *estimate_pls* function in the *seminar* package. The partial least squares method was preferred due to its ability to handle complex models and its robustness to violations of normality assumptions. We performed a bootstrap resampling procedure with 10,000 iterations to obtain robust parameter estimates and compute 95% CIs. The bootstrapped model was plotted to visualize the estimates and their 95% CIs.

## Results

Of 607 participants who completed the survey, 29.9% (n=182) used ChatGPT at least once per month, 26.1% (n=158) used it weekly, 24.5% (n=149) accessed it more than once per week, and 19.4% (n=118) interacted with it almost daily. A substantial portion of the participants held at least a high school diploma (n=204, 33.6%), and the majority had a bachelor’s degree (n=262, 43.1%). The primary motivations for participants to use ChatGPT were for acquiring information (n=219, 36%), amusement (n=203, 33.4%), and addressing problems (n=135, 22.2%). Some participants used it for health-related inquiries (n=44, 7.2%), while a few others (n=6, 1%) used it for miscellaneous activities such as brainstorming, grammar verification, and blog content creation. Table 2 shows the factor loading of the latent constructs in the model.

The model explained 2% and 64.6% of the variance in “satisfaction” and “trust,” respectively. Reliability estimates, as shown in Table 3, indicated high levels of internal consistency for all 5 latent variables, with Cronbach α and ρ values exceeding the recommended threshold of 0.7. The average variance extracted for the latent variables also exceeded the recommended threshold of 0.5, indicating that these variables are well-defined and reliable. Based on the root mean square error of approximation (RMSEA) fit index, our PLS-SEM model demonstrates a good fit for the observed data. The calculated RMSEA value of 0.07 falls below the commonly accepted threshold of 0.08, indicating an acceptable fit. The RMSEA estimates the average discrepancy per degree of freedom in the model, capturing how the proposed model aligns with the population covariance matrix. With a value below the threshold, it suggests that the proposed model adequately represents the relationships among the latent variables. This finding provides confidence in the model’s ability to explain the observed data and support the underlying theoretical framework.

Table 4 shows the estimated paths in our model. Hypothesis 1 postulated that as the perceived workload of using ChatGPT increases, user trust in ChatGPT decreases. Our analysis indicated a negative estimate for the path from workload to trust (–0.047). However, the *T* statistic (–1.674) is less than the critical value, and the 95% CI straddles 0 (–0.102 to –0.007), suggesting that the effect is not statistically significant. Therefore, we do not have sufficient evidence to support hypothesis 1.

Hypothesis 2 stated that perceived workload is negatively correlated with user satisfaction with ChatGPT. The results supported this hypothesis, as the path from workload to satisfaction showed a negative estimate (–0.142), a *T* statistic (–3.416) beyond the critical value, and a 95% CI (–0.223 to –0.061).

The data confirmed this relationship for hypothesis 3, which proposed a positive correlation between satisfaction with ChatGPT and trust in ChatGPT. The path from satisfaction to trust had a positive estimate (0.165), a *T* statistic (4.478) beyond the critical value, and a 95% CI (0.093-0.237).

**Figure 2:**
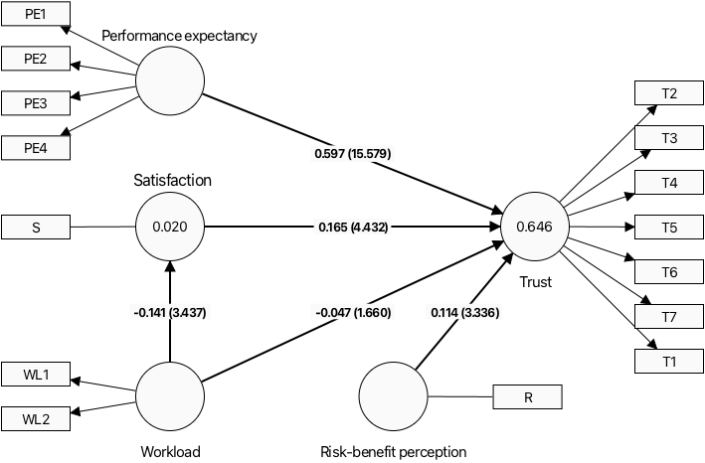
The significant paths connecting trust (T) in ChatGPT, performance expectancy (PE), satisfaction (S), workload (WL), and risk-benefit perception (R). T1 through T7: factors for trust; PE1 through PE4: factors for performance expectancy; and WL1 and WL2: factors for workload. The inner model shows the path coefficient and T statistic values.

Hypothesis 4 suggested that user performance expectations of ChatGPT increase with their trust in the technology. The analysis supported this hypothesis. The path from performance expectancy to trust displayed a positive estimate (0.598), a large *T* statistic (15.554), and a 95% CI (0.522-0.672). Finally, we examined hypothesis 5, which posited that user trust in ChatGPT increases as their risk-benefit perception of using the technology increases. The path from risk-benefit perception to trust showed a positive estimate (0.114). The *T* statistic (3.372) and the 95% CI (0.048-0.179) indicating this relationship is significant, but the positive sign suggests that as the perceived benefits outweigh the risks, the trust in ChatGPT increases. Therefore, hypothesis 5 is supported. Figure 2 illustrates the structural model with all path coefficients.

**Table 2:** Bootstrapped loadings: model analysis estimates the relationship between various constructs and their indicators.

Bootstrapped loadings			Loadings		*T* statistic	95% CI
**Trust (T)**						
	T1	0.788		41.998		0.750-0.823
	T2	0.753		33.795		0.706-0.794
	T3	0.773		40.293		0.733-0.808
	T4	0.732		28.772		0.679-0.779
	T5	0.673		21.066		0.607-0.732
	T6	0.799		46.065		0.763-0.831
	T7	0.779		38.088		0.736-0.816
**Performance expectancy (PE)**						
	PE1	0.809		49.231		0.775-0.839
	PE2	0.733		29.360		0.681-0.779
	PE3	0.802		44.968		0.766-0.835
	PE4	0.777		34.198		0.729-0.818
**Workload (WL)**						
	WL1	0.856		28.883		0.789-0.905
	WL2	0.913		44.872		0.869-0.950

**Table 3:** Convergent reliability.

Construct	Cronbach α	ρ C	AVE^a^	ρ A
Performance expectation	0.787	0.862	0.610	0.610
Workload	0.729	0.870	0.771	0.968
Trust	0.876	0.904	0.575	0.880

^a^AVE: average variance extracted.

**Table 4:** Bootstrapped structural path estimates.

Direct path	Bootstrap mean standard estimate (SD)	*T* statistic	95% CI
Risk-benefit perception→trust	0.114 (0.034)	3.372	0.048 to 0.179
Performance expectancy→trust	0.598 (0.038)	15.554	0.522 to 0.672
Workload→satisfaction	–0.142 (0.041)	–3.416	–0.223 to –0.061
Workload→trust	–0.047 (0.028)	–1.674	–0.102 to 0.007
Satisfaction→trust	0.165 (0.037)	4.478	0.093 to 0.237

## Discussion

### Main Findings

This study represents one of the initial attempts to investigate how human factors such as workload, performance expectancy, risk-benefit perception, and satisfaction influence trust in ChatGPT. Our results showed that these factors significantly influenced trust in ChatGPT, with performance expectancy exerting the strongest association, highlighting its critical role in fostering trust. Additionally, we found that satisfaction was a mediator in the relationship between workload and trust. At the same time, a positive correlation was observed between trust in ChatGPT and the risk-benefit perception. Our findings align with the May 23, 2023, efforts and initiatives of the Biden-Harris Administration to advance responsible AI research, development, and deployment [27]. The Administration recognizes that managing its risks is crucial and prioritizes protecting individuals’ rights and safety. One of the critical actions taken by the administration is the development of the artificial intelligence risk management framework (AI RMF). The AI RMF builds on the importance of trustworthiness in AI systems and is a framework for strengthening AI trustworthiness and promoting the trustworthy design, development, deployment, and use of AI systems, contributing to the need for our research [28]. Our findings reveal the importance of performance expectancy, satisfaction, and risk-benefit perception in determining the user’s trust in AI systems. By addressing these factors, AI systems can be designed and developed to be more user-centric, aligning with the AI RMF’s emphasis on human-centricity and responsible AI.

### Workload and Trust in ChatGPT

Moreover, we found that reducing user workload is vital for enhancing user satisfaction, which in turn improves trust. This finding aligns with the AI RMF’s focus on creating AI systems that are equitable and accountable and that mitigate inequitable outcomes. Additionally, our research emphasizes the need for future exploration of other factors impacting user trust in AI technologies. Such endeavors align with the AI RMF’s vision of managing AI risks comprehensively and holistically, considering technical and societal factors. Understanding these factors is crucial for fostering public trust and enhancing the overall trustworthiness of AI systems, as outlined in the AI RMF [28].

This study also extends and complements existing literature. Consistent with the observed patterns in studies on flight simulators, dynamic multitasking environments, and cyberattacks [29-31], we also found that higher perceived workload in using ChatGPT led to lower levels of trust in this technology. Our findings align with the existing research indicating a negative correlation between workload and user satisfaction [32]. We observed that as the perceived workload of using ChatGPT increased, user satisfaction with the technology decreased. This outcome echoes the consensus within the literature that a high workload can lead to user dissatisfaction, particularly if the technology requires too much effort or time [33]. The literature reveals that perceived workload balance significantly influences job satisfaction in work organizations [25], and similar patterns are found in the well-being studies of nurses, where perceived workload negatively impacts satisfaction with work-life balance [34]. While this study does not directly involve the workplace environment or work-life balance, the parallels between workload and satisfaction are evident. Furthermore, our research parallels the study suggesting that when providing timely service, AI applications can alleviate perceived workload and improve job satisfaction [35]. ChatGPT, as an AI-powered chatbot, could potentially contribute to workload relief when it performs effectively and efficiently, thereby boosting user satisfaction.

### Satisfaction and Trust in ChatGPT

Our findings corroborate with existing literature, suggesting a strong positive correlation between user satisfaction and trust in the technology or service provider [23,24,26,36-38]. We found that the users who expressed higher satisfaction with ChatGPT were more likely to trust the system, strengthening the premise that satisfaction can predict trust in a technology or service provider. Similar to the study on digital transaction services, our research indicates that higher satisfaction levels with ChatGPT corresponded with higher trust in the AI system [37]. This suggests that when users are satisfied with the performance and results provided by ChatGPT, they tend to trust the technology more. The research on mobile transaction apps mirrors our findings, where we also discovered that satisfaction with ChatGPT use was a significant predictor of trust in the system [36]. This showcases the importance of ensuring user satisfaction in fostering trust using innovative technologies like AI chatbots. The study on satisfaction with using digital assistants, where a positive relationship between trust and satisfaction was observed [26], further aligns with our study. We also found a positive correlation between trust in ChatGPT and user satisfaction with this AI assistant.

### Performance Expectancy and Trust in ChatGPT

Our findings concerning the strong positive correlation between performance expectancy and trust in ChatGPT serve as an extension to prior literature. Similar findings have been reported in previous studies on wearables and mobile banking [39,40], where performance expectancy was positively correlated with trust. However, our results diverge from the observations of a recent study that did not find a significant impact of performance expectancy on trust in chatbots [41]. Moreover, the observed mediating role of satisfaction in the relationship between workload and trust in ChatGPT is a notable contribution to the literature. While previous studies have demonstrated a positive correlation between workload reduction by chatbots and trust, as well as between trust and user satisfaction [42-44], the role of satisfaction as a mediator between workload and trust has not been explored. Finally, the positive correlation between the risk-benefit perception of using ChatGPT and trust aligns with the findings of previous studies [45-47]. Similar studies on the intention to use chatbots for digital shopping and customer service have found that trust in chatbots impacts perceived risk and is affected by the risk involved in using chatbots [46,47]. Our study adds to this body of research by confirming the same positive relationship within the context of ChatGPT.

### Limitations

Despite the valuable insights provided by this study, limitations should be acknowledged. First, our research focused explicitly on ChatGPT and may not be generalizable to other AI-powered conversational agents or chatbot technologies. Different chatbot systems may have unique characteristics and user experiences that could influence the factors affecting trust. Second, this study relied on self-reported data from survey responses, which may be subject to response biases and limitations inherent to self-report measures. Participants’ perceptions and interpretations of the constructs under investigation could vary, leading to potential measurement errors. Third, this study was cross-sectional, capturing data at a specific point in time. Longitudinal studies that track users’ experiences and perceptions over time provide a more comprehensive understanding of the dynamics between trust and the factors investigated. Finally, the sample of participants in this study consisted of individuals who actively use ChatGPT, which may introduce a self-selection bias. The perspectives and experiences of nonusers or individuals with limited exposure to AI-powered conversational agents may differ, and their insights could provide additional valuable perspectives.

### Conclusions

This study examined the factors influencing trust in ChatGPT, an AI-powered conversational agent. Our analysis found that performance expectancy, satisfaction, workload, and risk-benefit perceptions significantly influenced users’ trust in ChatGPT. These findings contribute to understanding trust dynamics in the context of AI-powered conversational agents and provide insights into the factors that can enhance user trust. By addressing the factors influencing trust, we contribute to the broader goal of fostering responsible AI practices that prioritize user-centric design and protect individuals’ rights and safety. Future research should consider longitudinal designs to capture the dynamics of trust over time. Additionally, incorporating perspectives from diverse user groups and examining the impact of contextual factors on trust would further enrich our understanding of trust in AI technologies.

## Abbreviations

AI: artificial intelligence
AI RMF: artificial intelligence risk management framework
PLS-SEM: partial least squares structural equation modeling
RMSEA: root mean square error of approximation
TAM: technology acceptance model
UTAUT: unified theory of acceptance and use of technology

## Data Availability

The data sets generated and analyzed during this study are available from the corresponding author on reasonable request.
